# The Influence of No-Primer Adhesives and Anchor Pylons Bracket Bases on Shear Bond Strength of Orthodontic Brackets

**DOI:** 10.1155/2013/315023

**Published:** 2013-08-04

**Authors:** Andrea Scribante, Maria Francesca Sfondrini, Danilo Fraticelli, Paola Daina, Alessandra Tamagnone, Paola Gandini

**Affiliations:** Dipartimento di Scienze Clinico Chirurgiche, Diagnostiche e Pediatriche, Unità di Odontoiatria, Reparto di Ortognatodonzia, Università degli Studi di Pavia, Piazzale Golgi, 2-27100 Pavia, Italy

## Abstract

*Objective*. The aim of this study was to compare the shear bond strength (SBS) and adhesive remnant index (ARI) scores of no-primer adhesives tested with two different bracket bases. *Materials and Methods*. 120 bovine permanent mandibular incisors were divided into 6 groups of 20 specimens. Two brackets (ODP) with different bracket bases (anchor pylons and 80-gauge mesh) were bonded to the teeth using a conventional adhesive (Transbond XT) and two different no-primer adhesive (Ortho Cem; Heliosit) systems. Groups were tested using an instron universal testing machine. SBS values were recorded. ARI scores were measured. SEM microphotographs were taken to evaluate the pattern of bracket bases. Statistical analysis was performed. ANOVA and Tukey tests were carried out for SBS values, whereas a chi-squared test was applied for ARI scores. *Results*. Highest bond strength values were reported with Transbond XT (with both pad designs), Ortho Cem bonded on anchor pylons and Heliosit on 80-gauge mesh. A higher frequency of ARI score of “3” was reported for Transbond XT groups. Other groups showed a higher frequency of ARI score “2” and “1.” *Conclusion*. Transbond XT showed the highest shear bond strength values with both pad designs.

## 1. Introduction

Orthodontic brackets have to deliver an optimal orthodontic force, must be able to withstand the masticatory loads, and should be easily removed at the end of the treatment with no or minimal damage to the tooth surface [1, 2].

In order to enhance speed of orthodontic bonding procedure no-primer adhesives have been introduced. Previous studies showed similar [3] or slightly lower [4] shear bond strength values when compared with conventional adhesive systems.

In fact masticatory loads can be very variable, as often some occlusal zones are differently and asymmetrically stressed during chewing. It has been demonstrated that the symmetry of surface electromyography activity is not related to symmetry of occlusal contacts [5], so during chewing some zones can be subjected to higher interarch occlusal forces than others. Therefore, orthodontic appliances often have to withstand variable masticatory loads, thus causing unwanted appliances debonding [6].

Bond failure of brackets is frustrating for the practitioner, affects appliance efficiency, has an economic impact on the practice, and can significantly delay the treatment progress [7, 8]. One reason for this occurrence, in addition to the type of adhesives used, can be the different bond strength of the orthodontic bracket pad [9]. In fact the morphology of the base is an important variable for the retention of a bracket [10]. Authors suggested that the base design may improve penetration of the adhesive material [11], and the size of the base has been seen to be also an important factor [12].

To our knowledge, there are no studies that compared shear bond strength of different no-primer adhesives tested in combination with different bracket pads. It would be interesting to evaluate various no-primer orthodontic adhesives tested in combination with different bracket bases in order to appreciate if some combinations are particularly favourable for bonding.

Accordingly, the aim of the present investigation was to measure and compare shear bond strength and adhesive remnant index score of one conventional and two different no-primer adhesives tested in combination with two different bracket pad designs. The null hypothesis of the study was that there is no significant difference in shear bond strength values and debond locations among the various groups.

## 2. Material and Methods

One hundred and twenty freshly permanent extracted bovine mandibular incisors were collected from a local slaughterhouse and stored in a solution of 0.1% (wt/vol) thymol. The criteria for tooth selection included intact buccal enamel with no cracks caused by extraction and no caries. 

The teeth were cleansed of soft tissue, and cold-curing, fast-setting acrylic resin (Leocryl, Leone, Sesto Fiorentino, Italy) was funneled inside a stainless steel cylinder (15 mm diameter). Vaseline was used as separating medium between the metal cylinder and the acrylic resin. Subsequently, the root of the tooth was inserted in the resin so that the vestibular portion of the crown was parallel to the metal cylinder. After resin hardening, each tooth resulted oriented so that its labial surface was parallel to shearing force.

**Figure 1:**
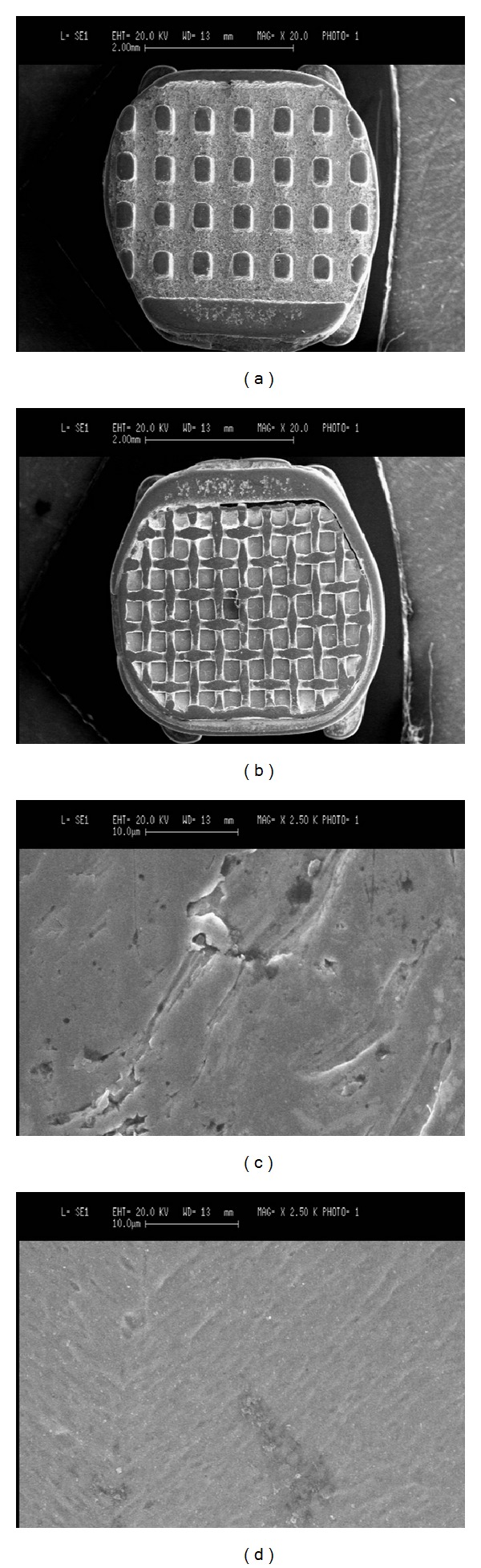
SEM microphotographs ((a), (b): ×20 and (c), (d): ×2500) of the two different bracket mesh designs tested: anchor pylons ((a), (c)) and 80-gauge mesh ((b), (d)).

Two different bracket mesh pad designs were investigated (Figures 1(a) and 1(b)): metal brackets with anchor pylons (ODP metal brackets with Anchor-Lock pad, Franklin, IN, USA) (Groups 1, 2, and 3) and metal brackets with 80-gauge mesh base (ODP metal brackets with Accu-Lock mesh, Franklin, IN, USA) (Groups 4, 5, and 6). Before preparing the specimens, scanning electron microscope photographs (×20 and ×2500) were taken using a scanning electron microscope (Stereoscan 360, Cambridge Instruments, Cambridge, England) to observe differences in bracket bases. 

Three different hybrid adhesive systems were tested (Table 1): a conventional orthodontic adhesive (Transbond XT, 3M Unitek, Monrovia, CA, USA) and two no-primer adhesives (Ortho Cem, FGM, Joinville, Brazil; Heliosit, Ivoclar Vivadent, Schaan, Liechtenstein).

Before bonding, the labial surface of each incisor was cleaned for 10 seconds with a rubber on low-speed handpiece with a mixture of water and fluoride-free pumice (Pomice Krugg, Krugg Spa, Buccinasco, MI, Italy). The enamel surface was rinsed 10 seconds with water and then dried with an oil-free air stream.

Enamel etching was conducted with 37% phosphoric acid gel (3M Unitek, Monrovia, CA, USA) for 30 seconds, followed by thorough washing and drying. 

In control groups (1 and 4) a thin layer of primer (Transbond XT, 3M Unitek, Monrovia, CA, USA) was applied on the etched enamel, and then the brackets were bonded with a resin (Transbond XT, 3M Unitek, Monrovia, CA, USA) near the center of the facial surface of the teeth (etching + priming resin + composite resin). 

In experimental Ortho Cem (2 and 5) and Heliosit (3 and 6) groups, the composite resin was applied on bracket base and bonded directly after etching, without primer application, following manufacturers' instructions (etching + composite resin).

Subsequently brackets of all groups were squeezed with sufficient pressure to express excess adhesive, which was removed with a scaler from the margins of the bracket base. Brackets were then light-cured with a visible light-curing unit (Ortholux XT, 3M Unitek, Monrovia, CA, USA) for 10 seconds on the mesial side of the bracket and for 10 seconds on the distal side (total cure time 20 seconds). After bonding, all samples were stored in thymol at room temperature for 24 hours and then tested in a shear mode on a universal testing machine (Model 4301, Instron, Canton, MA, USA).

### 2.1. Shear Bond Strength Testing

Specimens were secured in the lower jaw of the machine so that the bonded bracket base was parallel to the direction of the shear force.

Specimens were stressed in an occlusogingival direction (crosshead speed of 1 mm per minute) as in previous studies [1, 13]. The maximum load necessary to debond or initiate bracket fracture was recorded in newtons (N). Subsequently, values were converted into megapascals (MPa) as a ratio of newtons to surface area of the bracket. 

### 2.2. Optical Microscope Examination

After bond failure, both enamel surfaces and bracket bases were examined under an optical microscope (Stereomicroscope SR, Zeiss, Oberkochen, Germany) at 20x magnification, and the adhesive remnant index (ARI) score was recorded to assess the amount of adhesive left on the enamel surface [14]. The ARI scores were used as a more complex method of defining bond failure site among the enamel, the adhesive, and the bracket base. This scale ranges from 0 to 3. A score of 0 indicates no resin remaining on the tooth in the bonding area; 1 indicates less than half of the resin remaining on the tooth; 2 indicates more than half of the resin remaining on the tooth; and 3 indicates all resin remaining on the tooth, with a distinct impression of the bracket base. 

### 2.3. Statistical Analysis

Statistical analysis was performed with Stata 9.0 software (Stata, College Station, TX). Descriptive statistics including the mean, standard deviation, median, and minimum and maximum values were calculated for all groups. Normality of the data was assessed with Kolmogorov and Smirnov test. An analysis of variance (ANOVA) test was applied to determine whether significant differences in debond values existed among the groups. The Tukey test was used as *post hoc*. The chi-square test was used to determine significant differences in the ARI scores among the different groups. Significance for all statistical tests was predetermined at *P* < 0.05.

## 3. Results

**Figure 2:**
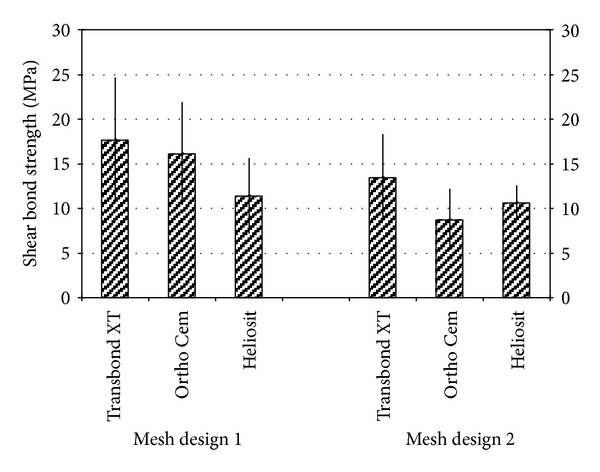
Shear bond strength values of the different groups.

Descriptive statistics for the shear bond strength (MPa) of the different groups are presented in Table 2. ANOVA showed the presence of significant differences among the various groups (*P* < 0.0001). *Post hoc* test showed that when testing brackets with anchor pylons, Transbond XT (Group 1) and Ortho Cem (Group 2) showed no significant difference between them (*P* > 0.05), and both exhibited significantly higher shear bond strength values (*P* < 0.05) than Heliosit (Group 3). On the other hand, when testing brackets with 80-gauge mesh base, Transbond XT (Group 1) and Heliosit (Group 3) presented no significant difference between them (*P* > 0.05), and both expressed significantly higher shear bond strength values than Ortho Cem (Group 2), as reported in Figure 2.

When considering the two different bracket bases tested (Table 3), brackets with anchor pylons (Groups 1, 2, and 3) showed significantly higher shear bond strength values than brackets with 80-gauge mesh base (Groups 4, 5, and 6) (*P* = 0.0013). In particular, when testing Transbond XT and Heliosit no significant differences were found when comparing shear bond strength of the two different bracket bases (*P* > 0.05), whereas for Ortho Cem a significant reduction in bond strength values (*P* < 0.001) was reported when testing brackets with 80-gauge mesh base. 

The results of ARI scores are presented in Table 4. The chi-square test reported a higher frequency of ARI score of “3” for groups 1 and 4 (*P* < 0.05) that showed no significant difference between them (*P* > 0.05). Other groups reported a higher frequency of ARI score “2” (Groups 3, 5, and 6) and “1” (Group 2).

SEM microphotographs of the two different pad designs (Figure 1) showed that the anchor pylons base showed a rougher surface than the 80-gauge base that presented a smoother surface.

## 4. Discussion

The null hypothesis of the study has been rejected. Brackets in the oral cavity are subjected to variety of forces. Bond strength is influenced by various factors like the surface area, conditioning procedures, type of adhesive used, bracket base design, the treatment of the bracket base, and protocol followed during bonding [15]. When evaluating scanning electron microphotographs of the recessions of the bracket bases, the anchor pylons base (Figure 1(a)) showed a narrower surface pattern (Figure 1(c)) than did the 80-gauge mesh base (Figure 1(b)) that showed a smoother surface design. When considering shear strength of the two different bracket bases tested, brackets with anchor pylons (Groups 1, 2, and 3) showed significantly higher shear bond strength values than brackets with 80-gauge mesh base (Groups 4, 5, and 6). Actually in the literature there are no studies that evaluated the effect of anchor pylons bracket bases on shear bond strength. In fact previous investigations [9, 11, 16] indicated a strong relationship between the retention capability and the base structure of orthodontic brackets. To enhance the retention of the adhesive to the metal base of orthodontic brackets, various chemical and mechanical retentive designs have been suggested [17]. Bases fused with metallic or ceramic particles [18], metal plasma-coated pads [19], and laser-structured bases [20] have been introduced. Moreover, mechanical retention was enhanced by enlarging the size of the base, by placing undercuts in the cast bracket bases, by welding different diameter mesh wires to the bracket base, as well as incorporating different designs in the mesh itself [12, 17]. The morphology of the base design may improve the penetration of the adhesive material [10]. Moreover, in the present investigation, when evaluating scanning electron microphotographs of the two different pad designs (Figure 1), the anchor pylons base (Figure 1(a)) showed a rougher surface (Figure 1(c)) than the 80-gauge base (Figure 1(b)) that presented a smoother surface (Figure 1(d)). As in previous investigations that tested different devices [9, 11] the pad with narrower grooves and undercuts should improve resin adhesion on a bracket base. A possible explanation for the results could be that changing the surface pattern allows increased base roughness and high bond strength.

In particular, in the present investigation, when testing Transbond XT and Heliosit, no significant differences were found when comparing shear bond strength of the two different bracket bases, whereas for Ortho Cem a significant reduction in bond strength values was reported when testing brackets with 80-gauge mesh base, thus suggesting that also adhesives played an important role in determining shear strength. Resin type might be of great effect, but also other factors should be considered, such as type, size, and distribution of particles. In fact in the present investigation three different adhesive systems were tested. The compositions are illustrated in Table 1. Conventional adhesive system (Transbond XT) presented the highest percentage (70–80 wt%) in filler content and the lowest values of resin (15–30 wt%). When evaluating the two no-primer adhesives, Ortho Cem reported less filler (45–65 wt%) and more resin (35–50 wt%) than conventional adhesive system. Heliosit showed the lowest filler percentage (14 wt%) and the highest resin percentage (85 wt%).

Brackets with anchor pylons pad performed higher shear strength with Transbond XT conventional adhesive system (with priming) and with Ortho Cem no-primer adhesive, whereas the lowest values were reported with Heliosit no-primer adhesive, thus indicating that with these devices the filler would not to be too low.

On the other hand brackets with 80-gauge mesh base presented higher shear strength with Transbond XT conventional adhesive system (with priming) and with Heliosit no-primer adhesive, thus indicating that with these devices adhesion is not influenced by the filler content but by resin type.

Transbond XT (with primer) and Heliosit (no-primer) have been extensively tested [4, 21], but only Transbond XT has been tested with different bracket pad designs [9, 10, 17]. No studies previously evaluated bond strength of Ortho Cem (no-primer) adhesive system. 

The analysis of the shear bond strength indicates that all the adhesive systems and both pad designs tested in the present investigations provided clinically acceptable bond force levels (6–8 MPa) suggested by Reynolds [22].

In the present study bovine teeth were used. Although these teeth differ from human ones in size and geometry, previous reports showed that bovine and human enamel are similar in their physical properties, composition, and bond strengths. Therefore permanent and deciduous bovine enamel has been demonstrated to be a reliable substitute for human enamel in bonding studies [23–25]. 

Finally the ARI scores were recorded. Groups 1 and 4 (conventional adhesive system with primer) presented a higher frequency of ARI score of “3.” Other groups (no-primer adhesives) reported a higher frequency of ARI score “2” (Groups 3, 5, and 6) and “1” (Group 2), thus showing that ARI scores seem not to be influenced by pad design but mainly from adhesive type. In the literature previous reports showed contradicting results. Different previous studies showed an insignificant [26, 27] or significant [28] influence of adhesive system on ARI scores. Moreover, both insignificant [10, 17, 29] and significant [11] effects of base design on ARI scores have been previously reported. This is probably due to the different materials and study design present in the various investigations.

Further studies are needed to complete and deepen the preliminary results of the present report about shear bond strength and ARI scores of no-primer adhesives.

## 5. Conclusions

When testing brackets with anchor pylons, Transbond XT and Ortho Cem showed no significant difference between them, and both exhibited significantly higher shear bond strength values than Heliosit. When testing brackets with 80-gauge mesh base, Transbond XT and Heliosit presented no significant difference between them, and both expressed significantly higher shear bond strength values than Ortho Cem.

When testing Transbond XT and Heliosit no significant differences were found when comparing shear bond strength of the two different bracket bases, whereas for Ortho Cem a significant reduction in bond strength values was reported when testing brackets with 80-gauge mesh base.

A higher frequency of ARI score of “3” is reported for Transbond XT groups that showed no significant difference between them. Other groups (no-primer adhesives) reported a higher frequency of ARI score “2” and “1.”

## Figures and Tables

**Table 1:** Composition (wt%) of the three different adhesives tested.

Adhesive	Resin	Filler	Additional contents
Transbond XT	Bisphenol A diglycidyl ether dimethacrylate (10–20 wt%) Bisphenol A bis(2-hydroxyethyl ether) dimethacrylate (5–10 wt%)	Silane-treated quartz (70–80 wt%)	Dichlorodimethylsilane reaction product with silica (<2 wt%)

Ortho Cem	Bisphenol A diglycidyl ether methacrylate (Bis-GMA) (25–35 wt%) Triethylene glicol dimethacrylate (TEGDMA) (10–15 wt%) Methacrylated phosphate monomer (>2 wt%)	Silane treated silicon dioxide (45–60 wt%)	Camphorquinone (<1 wt%) Sodium fluoride (>1 wt%)

Heliosit	Bis-GMA and decanediol dimethacrylate (85 wt%)	Highly dispersed silicon dioxide (14 wt%)	Catalysts and stabilizers (1 wt%)

**Table 2:** Descriptive and analytical statistics of the six groups tested (MPa).

Groups	Adhesive	Mesh	*n *	Mean	SD	Min	Median	Max	Tukey*
1	Transbond XT	Anchor pylons	20	17.67	6.90	6.16	16.29	31.19	A
2	Ortho Cem	Anchor pylons	20	16.10	5.76	7.15	16.41	25.96	A
3	Heliosit	Anchor pylons	20	11.35	4.20	4.29	10.60	22.24	C, B
4	Transbond XT	80-gauge mesh base	20	13.78	4.95	7.49	11.57	23.12	A, C
5	Ortho Cem	80-gauge mesh base	20	8.31	3.52	4.00	7.72	16.27	B, D
6	Heliosit	80-gauge mesh base	20	10.64	1.89	7.60	10.81	13.60	C, D

*Tukey grouping. Means with the same letters are not significantly different.

**Table 3:** Descriptive and analytical statistics of the comparison between anchor pylons and 80-gauge mesh base brackets (MPa).

Groups	Mesh	*n *	Mean	SD	Min	Median	Max	Significance*
1, 2, 3	Anchor pylons	60	14.96	6.22	4.29	13.30	31.19	A
4, 5, 6	80-gauge mesh base	60	11.60	4.42	4	10.79	23.12	A

*Statistical grouping. Means with the same letters are not significantly different.

**Table 4:** Frequency distribution of ARI scores in the different groups.

Groups	Adhesive	Mesh	*n *	ARI = 0	ARI = 1	ARI = 2	ARI = 3
1	Transbond XT	Anchor pylons	20	0 (0%)	3 (15%)	5 (25%)	12 (60%)
2	Ortho Cem	Anchor pylons	20	1 (5%)	11 (55%)	5 (25%)	3 (15%)
3	Heliosit	Anchor pylons	20	0 (0%)	8 (40%)	11 (55%)	1 (5%)
4	Transbond XT	80-gauge mesh base	20	0 (0%)	0 (0%)	3 (15%)	17 (85%)
5	Ortho Cem	80-gauge mesh base	20	0 (0%)	3 (15%)	13 (65%)	4 (20%)
6	Heliosit	80-gauge mesh base	20	0 (0%)	3 (15%)	14 (70%)	3 (15%)
